# Quantum semantics of text perception

**DOI:** 10.1038/s41598-021-83490-9

**Published:** 2021-02-18

**Authors:** Ilya A. Surov, E. Semenenko, A. V. Platonov, I. A. Bessmertny, F. Galofaro, Z. Toffano, A. Yu. Khrennikov, A. P. Alodjants

**Affiliations:** 1 ITMO University, St. Petersburg, Russia 197101; 2 Politecnico Milano, Italy Free University of Bozen, 39100 Bozen, Italy; 3 Laboratoire des Signaux et Systemes - L2S (UMR8506) - CNRS, Universite Paris-Saclay, Paris, France; 4 International Center for Mathematical Modeling in Physics and Cognitive Sciences, Linnaeus University, 351 95 Växjö, Sweden

**Keywords:** Physics, Quantum physics, Quantum information

## Abstract

The paper presents quantum model of subjective text perception based on binary cognitive distinctions corresponding to words of natural language. The result of perception is quantum cognitive state represented by vector in the qubit Hilbert space. Complex-valued structure of the quantum state space extends the standard vector-based approach to semantics, allowing to account for subjective dimension of human perception in which the result is constrained, but not fully predetermined by input information. In the case of two distinctions, the perception model generates a two-qubit state, entanglement of which quantifies semantic connection between the corresponding words. This two-distinction perception case is realized in the algorithm for detection and measurement of semantic connectivity between pairs of words. The algorithm is experimentally tested with positive results. The developed approach to cognitive modeling unifies neurophysiological, linguistic, and psychological descriptions in a mathematical and conceptual structure of quantum theory, extending horizons of machine intelligence.

## Introduction

Volumes of textual data, piling beyond capacity of human cognition, motivate development of automated methods extracting relevant information from corpuses of unstructured texts. As ensuring relevance requires prognosis of the user’s judgment, effective algorithms are bound, in some form, to simulate human-kind linguistic practice. This is an unsolved challenge, complexity of which was recognized long before computer age^1–4^. Now, with reading and writing texts turned into a massive and influencing part of creative human behavior, the problem is brought to the forefront of information technologies. Harnessing of human language skills is expected to bring machine intelligence to a new level of capability^5–7^.

As integral part of human cognition, natural language invites correspondingly integral modeling approach^8–13^. This is what we describe in this work. Our method of modeling, based on quantum-theoretic conceptual and mathematical structure, is common for various kinds of behavior including natural language^14^.

### Quantum-inspired cognitive modeling

Quantum theory reflects intrinsically uncertain, subjectively-contextual logic of human decision making allowing it to capture inherently human aspects of cognition and behavior such as individual unpredictability, associative irrational logic and cognition fallacies, emergent collective behaviors and others^14–17^. Complex nature of these phenomena makes them problematic to account with classical reductionist approach. Still, rational models of human choice developed in the era of mechanistic worldview hold as important limiting cases of individual and collective behavior^18^.

In general, probabilistic regularities of human behavior do not fit in a single-context Kolmogorovian probability space^19,20^; their description requires multi-context probability measure supplemented by transition rules between different contexts. Such measure is provided by quantum theory where the required contextual probability calculus is based on the notion of quantum state^21–25^. This allows to account for contextual cognitive and behavioral phenomena by simple and quantitative models reviewed in^15,26,27^.

### Advantage of quantum theory in language modeling

In natural language, quantum-like properties of human decision making manifest most clearly. By design, words of natural language are multifunctional, so that frequently used words, e.g. pad, have wide distributions of potential meanings^28^; only accommodation in a particular textual environment narrows this distribution to some extent. Still, a reader or listener puts it to his or her personal context that can alter the intended meaning dramatically^29,30^. For decades, principles of this sense-making process were addressed by traditional linguistics mostly by qualitative explanatory models^1,4,31,32^, cf.^33^; the modern practice-oriented paradigm shift was demanded by information technologies industry near the turn of the century^34–36^.

Quantum theory allows to describe semantic function of language quantitatively. In short, semantic fields of words are represented by superposition potentiality states, actualizing into concrete meanings during interaction with particular contexts. Creative aspect of this subjectively-contextual process is a central feature of quantum-type phenomena, first observed in microscopic physical processes^37,38^.

Deep similarity between quantum physical processes and cognitive practice of humans is a fundamental advantage of quantum approach in natural language modeling. This similarity allows to use quantum theory to reason sensibly about vector-space representation of semantics and probabilistic nature of observable textual events; crucially, this quantum-theoretic conceptual structure is expressed in strict mathematical framework allowing direct connection with measurable quantities^15^, ch.7. In accord, this makes a powerful navigator in space of behavioral and linguistic models as discussed in more detail in “Discussion” section.

#### Quantum approach to information retrieval

Quantitative models of natural language are applied in information retrieval industry as methods for meaning-based processing of textual data. As shown above, quantum modeling approach has unique advantage in addressing this challenge.

Quantum models, essentially, extend a standard vector representation of language semantics to a broader class of objects used by quantum theory to represent states of physical systems^39^. This allows to build explicit and compact cognitive-semantic representations of user’s interest, documents, and queries, subject to simple familiarity measures generalizing usual vector-to-vector cosine distance. The result is more precise estimation of subjective relevance judgments leading to better composition of search result pages^40–43^.

### This paper

Despite many promising results, quantum approach to human cognition and language modeling is still in a formation stage. A number of quantum-theoretic concepts and features stay unused, including complex-valued calculus of state representations, entanglement of multipartite systems, and methods for their analysis. Full employment of these notions in methods of machine text analysis is expected to start new generation of meaning-based information science^44^.

This paper addresses the above challenge by a model embracing both components just mentioned, namely complex-valued calculus of state representations and entanglement of quantum states. A conceptual basis necessary to this end is presented in “Neural basis of quantum cognitive modeling” section. This includes deeper grounding of quantum modeling approach in neurophysiology of human decision making proposed in^45,46^, and specific method for construction of the quantum state space. “Single-concept perception”, “Two-concept perception”, “Entanglement measure of semantic connection” sections describe a model of subjective text perception and semantic relation between the resulting cognitive entities.

In “Experimental testing” section the model is approbated in its ability to simulate human judgment of semantic connection between words of natural language. Positive results obtained on a limited corpus of documents indicate potential of the developed theory for semantic analysis of natural language.

## Results

### Neural basis of quantum cognitive modeling

#### Cognitive-physiological parallelism

In physical terms, control of the living system’s behavior is understood as electrochemical process occurring in an individual’s nervous system including $\sim$100 billion neuron cells interacting with each other via action potentials^47^. After initial formation by receptor cells, action potentials are transmitted through multilevel neuronal chains to the central nervous system and the brain where their transformation is observed by variety of physical means^48–50^. Resulting electrochemical excitations are transferred to the organism’s behavioral facilities by descending neural pathways.

Same phenomena can be described in information terms such that action potentials are considered as signals linking binary neural registers while total activity of the nervous system is referred to as psyche, cognition or mind^51,52^. In traditional psychology, activity of the mind is described verbally as dynamics of ideas, thoughts, motives, emotions, etc.^36,53^. Output of this dynamics controls observable behavior of an individual.

According to psycho-physiological parallelism^54^, modern cognitive science builds on fusion of physical and information descriptions outlined above, constituting complementary sides of the same phenomena^55–63^. In this approach, firing frequency of distributed ensembles of neurons functions as a code of cognitive algorithms and signals^64,65^. Detailed correspondence between these cognitive and physiological perspectives is established by dual-network representation of cognitive entities and neural patterns that encode them^59,66,67^.

**Figure 1:**
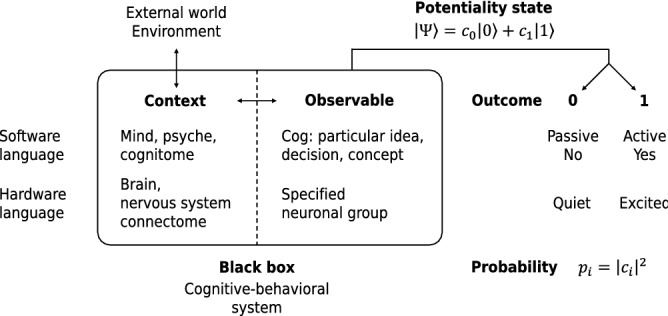
Quantum scheme of neuro-cognitive modeling. Cognitive and physiological terminologies reflect quantum-theoretic concepts (bold) in parallel way. In quantum approach, a cognitive-behavioral system is considered as a black box in relation to a potential alternative 0/1. Department of the black box responsible for the resolution of this alternative is observable, delineated from the context analogous to the Heienberg’s cut between the system and the apparatus in quantum physics. Relative to the dichotomic alternative 0/1, potential outcomes of the experiment are encoded by superposition vector state $\left| \Psi \right\rangle$ (1). If the experiment is performed, the system transfers to one of the superposed potential outcomes according to probabilities $p_i$.

#### Relation to quantum structure

The key provision of quantum modeling is that cognitive information is represented in discrete, i.e. quantized code. This is illustrated by all-or-none operation of a neuron cell: whereas the membrane’s voltage can take any value across continuous range, the meaningful signal, propagated further by action potential, is whether this voltage surpassed a certain discrete threshold or not^47^. On large scale, the discrete format is the only option meeting fundamental requirements of cognitive performance; in the alternative of continuous versus discrete encoding, only the latter allows for reliable transmission, storage and retrieval of information in the brain^68^.

In simplest discrete encoding, elementary units of cognition such as ideas, thoughts and decisions referred to as cogs^67^ are either active (1) or passive (0); in agreement with the neuro-cognitive correspondence these codes are associated with excited and quiet states of particular functional group of neurons^69^ realizing the cog. Probabilistic regularities of taking these (eigen)states in various potential contexts is an object of quantum modeling where alternatives 0 and 1 represent alternative states of a binary observable^45,70^.

Likelihood of activation of the considered cog in a particular situation is conditioned by its interaction with the rest of the cognitive system that in turn interacts with external world labeled in Fig. 1 as $<<$environment$>>$. Everything except the observed cog constitutes a set of experimental conditions called context^71^, so that delineation between the cog and this context represents a Heizenberg’s cut between the actualized conditions (classical side) and not yet actualized, i.e. potential state of the considered observable (quantum side)^72^.

Due to enormous number of uncontrolled degrees of freedom in the context (down to vacuum fluctuations of physical fields^73^, ch. 14), activation of the considered cog and the resulting cognitive-behavioral activity is fundamentally nondeterministic^74^. Corresponding probabilistic regularity is represented by potentiality state $\left| \Psi \right\rangle$ as indicated in the Fig. 1. Observable judgment or decision making records transition of a cognitive-behavioral system from state $\left| \Psi \right\rangle$ to a new state corresponding to the option actualized. (Since initially undefined observable and its context are parts of the same cognitive system, this transition is referred to as self-measurement. This simplest scheme is generalized to indirect and soft self-measurements by theory of quantum mental instruments^27,75^).

In this way, quantum approach allows to consider simple units of cognition while circumventing detailed description of the human’s mind and brain. At this level of modeling, numerous intricacies of human cognition are hidden, but continue to affect observable behavior (cf.^76^). Further sections illustrate this modeling approach on the process of subjective text perception.

#### Semantic-conceptual distinctions as cognitive basis

In our model, cognition of a subject is based on a set of linguistically expressed concepts, e.g. apple, face, sky, functioning as high-level cognitive units organizing perceptions, memory and reasoning of humans^77,78^. As stated above, these units exemplify cogs encoded by distributed neuronal ensembles^66^. Since the number of even single-word concepts in cognition of adult human is very large, each concept is passive most of the time, but may be activated by internal or external stimuli acquired e.g. from verbal or visual channels. This paper considers a particular class of such stimuli which are texts in natural language.

Composition of individual cognitive-conceptual structure is not fixed. Learning a concept apple, for example, amounts to configuring a specialized neuronal pattern that is reliably activated by appropriate complexes of visual, touch, taste, and smell signals^79^ and properly connected to other concepts^80^. This cognitive instrument allows an individual to distinguish apples from the background and use them at his or her discretion; this makes corresponding sensual information useful, i.e. meaningful for a subject^81–84^. Registry of such meaningful, or semantic, distinctions, usually expressed in natural language, constitutes a basis for cognition of living systems^85,86^. Alternatives of each semantic distinction correspond to the alternative (eigen)states of the corresponding basis observables in quantum modeling introduced above.

### Single-concept perception

Consider a single cognitive concept *X* in cognition of a subject, so that perception of a given text has potential to activate it (1) or not (0). Following^45,46^ we model this potentiality by two-dimensional vector

1 $$\begin{aligned} \left| \psi _x\right\rangle = c_0 \left| 0_x\right\rangle + c_1 \left| 1_x\right\rangle \end{aligned}$$

called qubit, where basis vectors $\left| 1_x\right\rangle$ and $\left| 0_x\right\rangle$ stand for potential outcomes of text perception that are active and passive states of a concept *X*, and $c_i$ are complex-valued amplitudes^87^. Probabilities with which alternative outcomes realize in potential perception experiment are defined as

2 $$\begin{aligned} p_i=\left| \left\langle i_x\right| \!\!\left. \, \psi _x\right\rangle \right| ^2=\left| c_i\right| ^2, \qquad p_0+p_1 = \left| c_0\right| ^2 + \left| c_1\right| ^2 = 1. \end{aligned}$$

Thus normalized vector (1) is a cognitive state representing the considered text relative to the concept *X* in cognition of a subject. In the process of perception, subjective cognitive basis $\left| 0_x\right\rangle$ and $\left| 1_x\right\rangle$ is analogous to measurement basis in quantum experiments, e.g. orientation of the magnets in the Stern-Gerlach experiment^88^. As in physics, superposition state refers to possible outcomes of the experiment which is not yet performed^89,90^. As in physics, cognitive superposition does not mean simultaneous coexistence of excited and quiet neural states realized by some sort of quantum magic^91^; rather, it accounts for a potential of transfer to new cognitive eigenstates in case the cognitive basis would be changed in a particular way^72,92^. (In our understanding, this parallel with physics is not yet complete since each mathematically possible transformation of basis $\{\left| 0_x\right\rangle ,\left| 1_x\right\rangle \}$ to some other $\{\left| 0_{x'}\right\rangle ,\left| 1_{x'}\right\rangle \}$ in cognitive case implies existence of linguistically expressed concept ${x'}$ together with the corresponding neuronal pattern, identifying of which is not obvious.)

Complex-valued amplitudes $c_i$ can be written in polar form

3 $$\begin{aligned} c_i = \sqrt{p_i} e^{i\phi _i}. \end{aligned}$$

Phase factors $e^{i\phi _i}$ generalize real-valued Euclidean vector space traditionally used for semantic modeling^78,93–95^ to complex-valued Hilbert space of quantum states (1). Phases $\phi _i$ do not enter probabilities (2) and therefore cannot be inferred from $p_i$; instead, values $\phi _i$ account for probabilities of different potential decisions related to $\{\left| 0\right\rangle _x,\left| 1\right\rangle _x\}$ by basis rotation. This allows quantum theory to account for subjectively-contextual nature of human cognition analogous to interference phenomena of wave physics^14,15^. As described in “Entanglement measure of semantic connection” section, in this work we report a novel use of the quantum phase parameters addressing semantic relation between a pair of qubit perception states of type (1).

#### Quantum semantic coherence

Preserving physical systems in superposition states (1) requires protection of the observable from interaction with the environment that would actualize one of the superposed potential states^96^. Similarly, preserving cognitive superposition means refraining from judgments or decisions demanding resolution of the considered alternative.

Cognitive coherence is necessary for adequate perception of indivisible blocks of sensory information constituting the essence of psychological gestalt^97–100^. Consider e.g. an instruction Disassemble the device after disconnecting it from the power outlet, semantics of which is to be evaluated for sentence as a whole. Relative to the observable decision $<<$do / not do$>>$, this requires holding the superposition state coherent until the end of the sentence (at least). Alternative strategy could be to collapse cognitive coherence after each, say, three words, followed by Bayesian update of judgment or decision probability, cf.^101^. This strategy, producing incorrect evaluation of semantics and correspondingly inadequate action, should be suppressed by natural selection in favor of quantum-like cognitive mechanics described above.

### Two-concept perception

In the following we focus on the case when text perception is based on two cognitive concepts labeled by words *A* and *B*; as shown in “Discussion” section, this seemingly unnatural situation is of direct practical interest. Distinctions $\left| 1_a\right\rangle , \left| 0_a\right\rangle , \left| 1_b\right\rangle , \left| 0_b\right\rangle$ generated by concepts *A* and *B* divide the semantic space into four orthogonal subspaces explicated in Table 1. Analogous to the single-concept case (1), joint cognitive potentiality of two considered concepts is represented by two-qubit state

4 $$\begin{aligned} \left| \Psi _{ab}\right\rangle = c_{00} \left| 00\right\rangle + c_{01} \left| 01\right\rangle + c_{10} \left| 10\right\rangle + c_{11} \left| 11\right\rangle , \end{aligned}$$

where complex-valued amplitudes are expanded as

5 $$\begin{aligned} c_{ij}=\sqrt{p_{ij}} e^{i \phi _{ij}}, \qquad \sum _{ij} p_{ij} = \sum _{ij} \left| c_{ij}\right| ^2=1. \end{aligned}$$

Analogous to single-concept case (3), $p_{ij}$ are probabilities with which four combinations of two binary distinctions encoded by words *A*, *B* and corresponding neuronal patterns would be activated in potential text perception experiment (Table 1).

**Table 1:** Two-dimensional Hilbert space of semantic categories defining text perception based on concepts *A* and *B*.

Concept activity state	Subspace of semantic Hilbert space
Both concepts A and B are active	$\left| 1_a\right\rangle \otimes \left| 1_b\right\rangle =\left| 11\right\rangle$
Concept A is active and concept B is passive	$\left| 1_a\right\rangle \otimes \left| 0_b\right\rangle =\left| 10\right\rangle$
Concept A is passive and concept B is active	$\left| 0_a\right\rangle \otimes \left| 1_b\right\rangle =\left| 01\right\rangle$
Both concepts A and B are passive	$\left| 0_a\right\rangle \otimes \left| 0_b\right\rangle =\left| 00\right\rangle$

**Figure 2:**
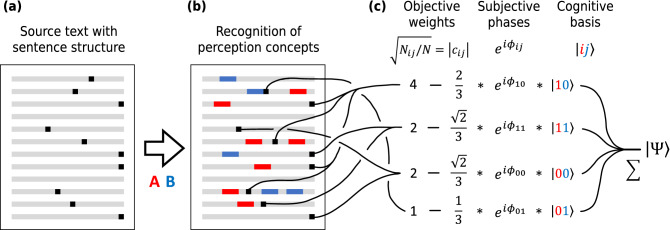
Text perception model: construction of the quantum cognitive state from the text substrate. Source document with sentences delimited by black squares (**a**) is perceived through binary distinctions expressed by concepts A and B. Presence of words associated with A and B (identified with neighboring sets, see Calculation of amplitudes) marked by red and blue (**b**) categorizes sentences to semantic subspaces defined by distinction states (Table 1). Number of sentences in each category defines absolute values of the amplitudes $|c_{ij}|=\sqrt{p_{ij}}$ in cognitive state $\left| \Psi \right\rangle$ (4). The model is finalized by supplementing the amplitudes with phase factors $e^{i \phi_{ij}}$ representing subjective dimension of text perception (**c**).

#### Calculation of probabilities

Whatever number of cognitive distinctions is used by subject, amplitudes $c_{i}$ in (1) or $c_{ij}$ in (4) are to be determined during the text perception. For the two-concept case, we model this process by the following algorithm visualized in Fig. 2:

1. Identify set of words $O_w$ which co-occur with word $w\in \{A,B\}$ in the same sentence of the text;
2. Classify sentences of the text in 4 categories corresponding to basis vectors of the semantic Hilbert space listed in Table 1 by presence of members of $O_w$. For example, sentence is assigned to class $\left| 01\right\rangle$ iff it contains no words from set $O_A$ and any number of words from set $O_B$;
3. For each $i,j\in \{0,1\}$ set $p_{ij}=N_{ij}/N$, where $N_{ij}$ is the number of sentences in each category and $N = \sum N_{ij}$ is total number of sentences in text.

The logic behind this algorithm is that sentences are treated as identically prepared instances of the text analyzed by subject, so that statistics of *N* recognition experiments is used to define amplitudes of state (4). This definition of amplitudes is by no means the only possible; it is chosen due to its sufficiency for the proof-of-principle demonstration pursued in this paper. For example, in the approach developed by Galofaro et al. semantic dimensions are extracted from the word co-occurrence matrix of the considered text, which allows to construct four-dimensional state of the form (4) reflecting interpretable semantics relations between the basis words^102^.

The above algorithm specifies only absolute values of the amplitudes $c_{ij}$, leaving their phase factors $\phi _{ij}$ undefined. This reflects intrinsically subjective nature of meaning-making perception process, result of which is not predefined by input information, but equally depends on semantic regularities of the considered perception system. This is further discussed in “Experimental testing” and “Discussion” sections.

### Entanglement measure of semantic connection

If the considered text is random word sample randomly divided to sentences by dots, then occurrences of any two words *A* and *B* in sentences are independent random variables so that

6 $$\begin{aligned} \dfrac{N_{01}}{N_{00}} = \dfrac{N_{11}}{N_{10}}, \end{aligned}$$

for any algorithm of sentence categorization. In the case of real-valued amplitudes, pure state (4) then reduces to a product of two factors

7 $$\begin{aligned} \left| \Psi \right\rangle =&\left( a_0\left| 0_a\right\rangle + a_1 \left| 1_a\right\rangle \right) \otimes \left( b_0\left| 0_b\right\rangle + b_1 \left| 1_b\right\rangle \right) = \left| \psi _a\right\rangle \otimes \left| \psi _b\right\rangle , \end{aligned}$$

where

$$\begin{aligned} a_0^2 = \dfrac{N_{00}+N_{01}}{N}, \quad a_1^2 = \dfrac{N_{10}+N_{11}}{N}, \quad b_0^2 = \dfrac{N_{00}+N_{10}}{N}, \quad b_1^2 = \dfrac{N_{01}+N_{11}}{N} \end{aligned}$$

and single qubit states $\left| \psi _a\right\rangle$ and $\left| \psi _b\right\rangle$ represent marginal cognitive models of text perceived through isolated conceptual distinctions *A* and *B*.

Impossibility of factorization (7) known as entanglement^103^ is a property of a compound state (4) in which subsystems have potential for coordinated resolution of uncertainties. Quantum entanglement between cognitive subspaces $\left| 00\right\rangle$, $\left| 01\right\rangle$, $\left| 10\right\rangle$, $\left| 11\right\rangle$ in (4) models semantic connection between concepts *A* and *B* as subjectively established by an individual recognizing the text. So defined semantic connection is ubiquitous in human cognition, where holistic entities are described not by individual signs but by compositions thereof^31,80^; description of this phenomenon in terms of quantum entanglement shows significant explanatory power^104–108^.

#### Concurrence

The amount of entanglement present in the pure two-qubit state (4) is quantified by deviation from factorization condition (7). In quantum information science, the corresponding measure called concurrence is defined as

8 $$\begin{aligned} \begin{aligned} Q = \left| \left\langle \Psi _{ab}\right| \hat{\sigma }_y \otimes \hat{\sigma }_y \left| \Psi ^*_{ab}\right\rangle \right| = 2 \left| c_{01}c_{10} - c_{00}c_{11} \right| , \qquad 0\le Q \le 1. \end{aligned} \end{aligned}$$

where $\hat{\sigma }_y$ are Pauli Y operators acting in single qubit subspaces *A*, *B* and * is complex conjugation^109^. Using $p_{ij}=N_{ij}/N$ as described in “Two-concept perception” section, polar expansion of amplitudes $c_{ij}$ (5)

9 $$\begin{aligned} c_{ij}=\sqrt{\dfrac{N_{ij}}{N}} e^{i \phi _{ij}} \end{aligned}$$

transforms expression (8) to

10 $$\begin{aligned} \begin{aligned} Q = 2\left| \dfrac{\sqrt{N_{01}N_{10}}}{N}e^{i (\phi _{01} + \phi _{10})}-\dfrac{\sqrt{N_{00}N_{11}}}{N}e^{i (\phi _{00} + \phi _{11})}\right| = \,\, & 2 \sqrt{\dfrac{N_{01}N_{10} + N_{00}N_{11}}{N^2} - 2 \dfrac{\sqrt{N_{01}N_{10}N_{00}N_{11}}}{N^2} \cos \Delta }, \\&\Delta = \phi _{01} + \phi _{10} - \phi _{00} - \phi _{11}. \end{aligned} \end{aligned}$$

Quantity (10) is computable from the number of sentences $N_{ij}$ in four semantic categories and a single four-phase difference $\Delta$. In the following, we use this latter inherently quantum-theoretical degree of freedom as a fitting parameter allowing to tune concurrence value for given count statistics $N_{ij}$. This feature reflects subjective aspect of text perception that is orthogonal to the objective count statistics of word’s co-occurrence in text.

Averaging over $\Delta$ nullifies the second summand under root in (10), making the resulting expression similar to the phi coefficient (mean square contingency)

11 $$\begin{aligned} C = \dfrac{N_{00}N_{11} - N_{10}N_{01}}{\sqrt{(N_{00}+N_{01})(N_{10}+N_{11})(N_{00}+N_{10})(N_{01}+N_{11})}}, \qquad -1\le C \le 1 \end{aligned}$$

measuring classical correlation between the two binary variables, i.e. correlation of co-occurrence of words *A*, *B* in the document’s sentences^110^. Numerator of expression (11), quantifying deviation of count statistics from classical factorization condition (6), can be obtained by replacement of amplitudes $c_{ij}$ in (8) by the corresponding probabilities (4). By additional phase dimensions $\phi _{ij}$, concurrence measure (8) generalizes classical correlation (11) to the quantum domain.

Concurrence value (10) defines maximal violation of Bell’s inequality also used to detect entanglement of two-qubit state (4) in quantum physics and informatics^87,111^. This relates the model of perception semantics developed in this paper with Bell-based methods for quantification of quantum-like contextuality and semantics in cognition and behavior^106,107,112,113^. Concurrence entanglement measure of the two-qubit cognitive state can be compared with quantification of semantic connection by Bell-like inequality introduced in^114^. Use of different Pauli operators in (8) may account for distinction between classical and quantum-like aspects of semantics^102^.

### Experimental testing

The semantics-detection method described in “Entanglement measure of semantic connection” section was tested for a pair of concepts *A*=website and *B*=promotion for probe documents listed in Table 2. Documents were estimated by 8 experts according to how well they answer a question “What is website promotion?”. Means of the obtained grades for each document is shown in the first column of Table 2. Standard deviation of expert’s grades for each document averaged for all documents is 1.6.

**Figure 3:**
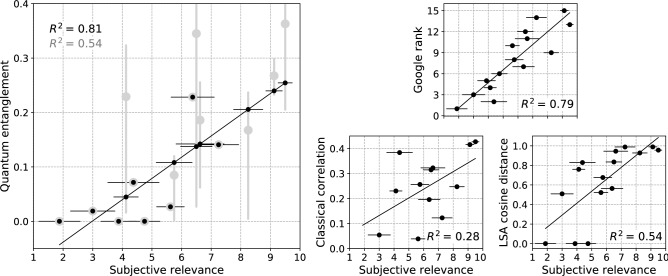
Left: semantic connection between concepts website and promotion quantified by concurrence entanglement measure (10) versus expert estimation of how well text answers the question $<<$What is website promotion?$>>$ for 15 probe documents. Gray bars show range of concurrence values accessible by tuning of quantum phases $\phi _{ij}$ in perception model of each document. Compared to the phase-randomized concurrence (gray circles), phase-tuned values (black dots) increase $R^2$ from 0.54 to 0.81. Top right: ranking of the probe documents by Google search for the query website promotion. Bottom right: classical correlation (11) and the LSA cosine distance between the same concepts (Methods). $R^2$ are determination coefficients. Horizontal axis, data and statistical error bars are common for all panels except three documents for which classical correlation is undefined. All data are given in Table 2.

For each document, the perception model (4) was built and used to calculate the concurrence measure (10) that is plotted versus expert’s estimation in Fig. 3, left panel. In cases when factor $\sqrt{N_{01}N_{10}N_{00}N_{11}}$ before $\cos \Delta$ is nonzero (documents 1,2,3,5,6,8,12), phase $\Delta$ allows to tune the concurrence value in the limits shown by gray bands; the phase-randomized values are shown by gray dots (data are given in Table 2). Phase $\Delta$ was set to minimize deviation of concurrence from the expert’s rank measured by determination coefficient $R^2$ of linear regression^110^; the resulting concurrence values are shown in the left panel of Fig. 3 by black dots. Starting from phase-randomized values (gray dots), this optimization increased $R^2$ from 0.54 to 0.81.

Remaining panels of Fig. 3 show alternative measures of semantic relation. Right bottom panels are classical binary correlation (11) and LSA cosine distance between words *A* and *B* (Methods) plotted versus the same expert’s estimation as the main panel. Corresponding determination coefficients 0.46 and 0.54 are inferior to the optimized quantum model. Top right panel of Fig. 3 shows ranking of the probe documents by Google search engine in response to query website promotion, used as estimator of semantic relation between the query words (Methods). The obtained determination coefficient $R^2=0.79$ is slightly inferior to that demonstrated by the optimized concurrence measure. Similar results are obtained for Russian language.

## Discussion

### The question of quantumness

Modeling of natural language, quantum and otherwise, aims to understand human language practice usually by reproducing it in machine-friendly algorithmic form. In contrast to cryptographical and computation algorithms of quantum information science, these algorithms are mostly designed for ordinary classical computers. $<<$Quantumness$>>$ of such algorithms is then usually cast to question. If the result of modeling is expressible in standard programming language, is there any significant reason to call such model quantum?

The answer to this question becomes evident by observing that encountering an effective model or algorithm by blind search is practically impossible. The space of possibilities is so enormous that finding a good solution requires general reason about where to look, what may work, and what surely can’t. For example, the neural network paradigm is obviously based on the brain’s working, while the very representation of information in binary code reflects Boolean logic observed in inert macroscopic processes. Similarly, ideas for modeling of non-deterministic phenomena can be borrowed from quantum theory that guides thought and suggests instruments. The resulting models of human behavior are quantum in the same way as ordinary computing is classic-mechanical and neural networks are biological in their origin. The term $<<$quantum$>>$ is retained, e.g., in the title of this paper as indication of its parent conceptual structure differing from the mainstream research.

### Quantum neuro-cognitive modeling

The modeling approach described above simulates conceptual human cognition responsible for language practice and decision making. It represents high-level counterpart of the neural-network models emulating human cognition on the level of individual brain cells^115,116^. Correspondence between these two approaches would allow for neural networks with interpretable internal operation, cf.^117–119^. This, in turn, is a way to build antropomorphic computational systems able of strong semantic computing – $<<$systems that know what is going on$>>$ and $<<$what they are doing$>>$^120,121^. Quantum approach to design of human-like semantic perception, the necessary part of such systems^122,123^, is illustrated by the model above.

Cognitive states formed in the process of perception of text are fully compatible with quantum theoretic analysis methods. In this way, concurrence measure of quantum entanglement is imported from quantum theory to the cognitive domain for free. The resulting model quantifies subjective familiarity between cognitive entities that is an essential in knowledge systems^36,124^. In texts, it allows to extract and quantify meaning relations between concepts, requested for semantic analysis of natural language data^125–127^. Simplicity and interpretability of the model, in accord with the positive results reported above, exemplifies advantage of quantum approach to cognitive modeling discussed in the beginning of this section.

### Relation to QBism

Principles of quantum neuro-cognitive modeling developed in “Neural basis of quantum cognitive modeling” section complement subjective interpretation of quantum theory (QBism) in which quantum theory constitutes a personalized instrument for probabilistic prognosis of individual experience^128–130^. Even though the latter is intrinsically subjective and associated with non-physical terms like consciousness and awareness, its brain-state representation is a part of physical world ruled by laws of neurophysics^47^. Akin to states of elementary particles in quantum physical laboratories, neurally encoded mental states can be both actual and potential, so that the former functions as a $<<$classical$>>$ experimental apparatus actualizing one of potential futures of its <<quantum>> part according to the laws of quantum theory (Fig. 1).

In that way, QBism is consistent with methodology of quantum neuro-cognitive modeling described above, cf.^116^. In the spirit of QBism, our model explicitly describes cognition of a $<<$user who is trying to make sense of that world$>>$^25,82,85,131–134^. In particular, it provides top-level counterpart for neurophysiological methods of revealing and quantifying cognitive relations like fMRI adaptation^135,136^ that can be used in semantic studies of human cognition^67,137–139^.

### Quantum phases and prediction power

Understanding of the phase parameters is a hard question in quantum cognitive and behavioral modeling. Possible approach to this problem is suggested by neurophysiological parallel of quantum cognitive modeling developed in “Results” section. According to this correspondence, quantum phases are phases of neural oscillation modes^65,140–142^, encoding cognitive distinctions represented by quantum qubit states as shown in Fig. 1, cf.^143^.

In cognitive perspective, complex-valued probability calculus of quantum modeling accounts for intrinsic subjectivity of semantics. While absolute values of perception-state amplitudes $c_{ij}$ reflect objective coincidence rates $N_{ij}$, phase factors $\phi _{ij}$ cannot be extracted from the text data. These factors depend on the individual perception system thereby representing subjective aspect of human cognition that is overlooked in other paradigms of semantic modeling^137,138^.

Post-factum fitting of phase data presented above is in line with the basic practice of quantum cognitive modeling^14,15^. In the present case, it constitutes finding of what the perception state should be in order to agree with the expert’s document ranking in the best possible way. Detailed analysis of this mechanism is subject for future study. Upgrading quantum decision model from descriptive to predictive status is possible by supplying it with quantum phase regularities encoding semantic stability of cognitive patterns^144,145^.

### Application to information retrieval

Immediate application of the developed model is information retrieval. Using subjective relevance judgment as observable for semantic connectivity can be seen as inverse of the basic objective of information retrieval science aiming to rank text documents according to the user’s needs. In this analogy, concepts *A* and *B* constitute a two-word search query, while semantic connection quantified by concurrence (10) calculated for each text document in the corpus is used to decide relevance the documents to the search query and to rank them in search result page.

In absence of other data, query $<<A \,\, B>>$ constitutes the only information about user’s interest available to the search engine. Internet search activity thereby realizes a distilled two-concept interaction hardly possible in human-to-human communication. In this situation, the two-concept perception model developed in “Two-concept perception” section is a model of the user’s cognition that a search engine provided with a query $<<A \,\, B>>$ may build for a given text.

### Scaling of semantics: from the bag of words to the bag of sentences and further

According to calculation of amplitudes described in “Results” section, cognitive model of the text (4) depends on its sentence structure. In particular, random shuffle of words and periods leads to factorization of state (4) and zero concurrence which reflects elimination of semantic connection. At the same time, calculation of amplitudes is not affected by shuffle of both sentences within text and words within sentences, so that subsequent calculation of concurrence as measure of semantic connection is also invariant to these operations. The algorithm thereby treats text as a bag of sentences which may be paralleled with a bag of words level of text analysis^146,147^. This specifies level of semantics that can be detected as entanglement between corresponding cognitive representations.

Sentence-level perception and semantic analysis described above can be scaled to paragraphs, chapters, whole texts, and even larger structures, addressing the problem of computational scalability^95,148,149^. For example, perception of the text as a bag of paragraphs can be accounted by exactly the same model that works with words and sentences. In that way, hierarchical semantic structure of information representation, typical to human cognition^9,150^, can be accessed.

## Materials and methods

**Table 2:** Results of testing the quantum model of semantic connection between concepts website and promotion for 15 probe documents. Documents are listed in order of their mean ranking by experts according to how well they answer the question $<<$What is website promotion?$>>$ (column 1). Column 2 shows value of concurrence measure of semantic entanglement (10) between words website and promotion, randomized in $\Delta$; for those documents where factor $\sqrt{N_{01}N_{10}N_{00}N_{11}}$ is nonzero, upper and lower bounds are shown. Columns 3–5 contain classical correlation (11), Google rank in response to the query website promotion and LSA cosine distance between the same words in 12 dimensions.

Expert estimation	Quantum entanglement (10)	Classical correlation (11)	Google rank	LSA cosine distance	Document
9.5	$$0.36\begin{array}{c} +0.10 \\ -0.15 \end{array}$$	0.43	3	0.96	How to promote your website online (for free!)
9.1	$$0.27\begin{array}{c} +0.03 \\ -0.04 \end{array}$$	0.42	1	0.99	How to promote your website: tips for digital domination
8.3	$$0.17\begin{array}{c} +0.07 \\ -0.16 \end{array}$$	0.25	7	0.93	How to promote your blog
7.3	0.14	0.12	2	0.99	7 Best techniques to promote your website for free
6.6	$$0.19\begin{array}{c} +0.07 \\ -0.12 \end{array}$$	0.32	5	0.95	33 Creative ways to promote your app for free
6.5	$$0.34\begin{array}{c} +0.14 \\ -0.32 \end{array}$$	0.31	4	0.84	Content promotion: how to balance organic results with paid ads
6.4	0.23	0.20	9	0.56	10 Social-media marketing strategies for companies
5.8	$$0.085\begin{array}{c} +0.04 \\ -0.08 \end{array}$$	0.26	8	0.68	The 11 golden rules of writing content for your website
5.6	0.04	0.04	6	0.52	Promotion (marketing) (Wikipedia article)
4.8	0	-	10	0	6 Free analytics tools to help you understand your competitor’s web traffic
4.4	0.07	0.38	14	0.83	27 of the best website designs to inspire you in 2020
4.1	$$0.23\begin{array}{c} +0.09 \\ -0.21 \end{array}$$	0.23	12	0.76	Internet branding (Wikipedia article)
3.9	0	–	11	0	How to start your own brand from scratch in 7 steps
3	0.02	0.05	13	0.51	Website (Wikipedia article)
1.9	0	–	15	0	The new age of content Darwinism (and how to apply it)

### Probe documents and concepts

Concepts *A* and *B* are taken to be concepts of natural language website and promotion. Logic behind this choice is that both concepts are to have well-defined standalone meanings different from that of their combination, so that texts which are relevant to any single of two concepts are irrelevant to the compound query. The pair website and promotion meets this requirement since both texts on the main meaning of promotion as marketing activity and texts on the main meaning of website as Internet entity are weakly relevant to one interested in website promotion. Experts were asked to estimate the degree of how much probe text answers the question $<<$What is website promotion?$>>$ by integers from 0 (does not answer) to 10 (perfect answer). The probe documents are listed in Table 2.

### Measurement of semantic observable

When interested in text perception a subject may browse articles and books for existing results. By all likelihood, encyclopedia articles on text and perception alone will not be very helpful; what’s needed is a text which describes how the two entities relate to each other. In other words, satisfying interest in text perception amounts to establishing semantic connection between terms text and perception. Based on that we consider relevance of a given text to the compound two-word query $<<A \,\, B>>$ estimated by subject as an observable factor quantifying of semantic connection between concepts *A* and *B* in this text. Namely, subjective relevance score ranging from 0 (minimal relevance) to 10 (maximal relevance) is linearly mapped to the range (10) of concurrence measure of semantic connection.

### Search engine as semantic estimator

In accord with the previous paragraph, reliability of linear regression between expert’s estimation and Google ranking ($R^2=0.79$ and p-value of $10^{-5}$. Similar results are observed for Yandex) supports the use of search engine as estimator of semantic relation. Namely, finding document *X* higher than document *Y* in a search engine $\alpha$ result page in response to the query $<<A \,\, B>>$ implies that semantic connection between words *A* and *B* is stronger in document *X* than in document *Y*. This performance of search engines reflects stable semantic patterns of their user’s cognition. Popular page ranking algorithms thus have potential to substitute real subjects in experimental semantic research, cf. e.g.^151^.

### Estimation of semantic relation by LSA cosine distance

Cosine distance measure used as estimator of semantic connection is produced from representation of target words in 12-dimensional latent semantic space constructed for each document^152,153^. Quantity shown in the right bottom panel of Fig. 3 is scalar product $-1\le \mathbf {w}_a \mathbf {w}_b\le 1$ of vectors representing query words *A* and *B*.
